# Proteomic analysis of effluents from perfused human heart for transplantation: identification of potential biomarkers for ischemic heart damage

**DOI:** 10.1186/1477-5956-10-21

**Published:** 2012-03-23

**Authors:** Hong Li, Jiyuan Li, Ying Wang, Tiande Yang

**Affiliations:** 1 Department of Anesthesiology, Xinqiao Hospital, Third Military Medical University, Chongqing 400037, China; 2 Chongqing Key Laboratory for Disease Proteomics, Chongqing 400038, China

**Keywords:** Proteome analysis, Human perfused heart, Ischemia

## Abstract

**Background:**

Biomarkers released from the heart at early stage of ischemia are very important to diagnosis of ischemic heart disease and salvage myocytes from death. Known specific markers for blood tests including CK-MB, cardiac troponin T (cTnT) and cardiac troponin I (cTnI) are released after the onset of significant necrosis instead of early ischemia. Thus, they are not good biomarkers to diagnose myocardial injury before necrosis happens. Therefore, in this study, we performed proteomic analysis on effluents from perfused human hearts of donors at different ischemic time.

**Results:**

After global ischemia for 0 min, 30 min and 60 min at 4°C, effluents from five perfused hearts were analyzed respectively, by High performance liquid chromatography-Chip-Mass spectrometry (HPLC-Chip-MS) system. Total 196 highly reliable proteins were identified. 107 proteins were identified at the beginning of ischemia, 174 and 175 proteins at ischemic 30 min and ischemic 60 min, respectively. With the exception of cardiac troponin I and T, all known biomarkers for myocardial ischemia were detected in our study. However, there were four glycolytic enzymes and two targets of matrix metalloproteinase released significantly from the heart when ischemic time was increasing. These proteins were L-lactate dehydrogenase B(LDHB), glyceraldehyde-3-phosphate dehydrogenase, glucose-6-phosphate isomerase (GPI), phosphoglycerate mutase 2 (PGAM2), gelsolin and isoform 8 of titin. PGAM2, LDHB and titin were measured with enzyme-linked immunosorbent assays kits. The mean concentrations of LDHB and PGAM2 in samples showed an increasing trend when ischemic time was extending. In addition, 33% identified proteins are involved in metabolism. Protein to protein interaction network analysis showed glycolytic enzymes, such as isoform alpha-enolase of alpha-enolase, isoform 1 of triosephosphate isomerase and glyceraldehyde-3-phosphate dehydrogenase, had more connections than other proteins in myocardial metabolism during ischemia.

**Conclusion:**

It is the first time to use effluents of human perfused heart to study the proteins released during myocardial ischemia by HPLC-Chip-MS system. There might be many potential biomarkers for mild ischemic injury in myocardium, especially isoform 8 of titin and M-type of PGAM2 that are more specific in the cardiac tissue than in the others. Furthermore, glycolysis is one of the important conversions during early ischemia in myocardium. This finding may provide new insight into pathology and biology of myocardial ischemia, and potential diagnostic and therapeutic biomarkers.

## Introduction

Ischemic Heart Disease (IHD) is the most common cause of death and a major cause of hospital admissions in most Western countries [1]. The diagnosis of IHD is based on particular symptoms, an electrocardiogram, an X-ray of the chest and blood tests. Reasonably specific markers for blood tests including creatine kinase muscle/brain isoform (CK-MB), cardiac troponin T (cTnT) and cardiac troponin I (cTnI) are released after the onset of significant necrosis instead of early ischemia, and they all require a level of myocardial necrosis to prompt their release from myocytes before they can be detected. Those biomarkers are impossible to be detected at early stage of ischemia and the diagnosis of IHD is often ambiguous. On the other hand, it is impossible to salvage dead myocytes at the stage of necrosis when specific markers such as CK-MB, cTnT and cTnI are detected. Thus, biomarkers detectable before the onset of significant necrosis would be more important than those in current use. Evidence has shown that markers released upon initiation of ischemia alone may exist [2-4]. If it is true, such markers would offer the opportunity for early diagnosis of IHD before permanent myocyte damage occurs, which will allow possible salvage of the myocardium by timely reperfusion. However, it is very difficult to discover a novel biomarker by screening the entire proteome of plasma from the IHD patients [4,5]. This is because there are many highly abundant proteins present in the serum or plasma, which will mask biomarkers, presumably the less abundant proteins. Proteomic analysis of the plasma from patients with acute coronary syndromes (ACS) revealed only five differentially expressed proteins, all of which were highly abundant plasma proteins [6]. Thus, it is very important to eliminate the interference by highly abundant plasma proteins. Isolated perfused heart effluent is a novel model for protein biomarker discovery. This model dispenses with most highly abundant proteins in blood [7]. In our study, effluents from human perfused heart for transplantation at different ischemic time points were collected for proteomic analysis to identify potential ischemic biomarkers.

## Materials and methods

### Effluent collection and concentration

The study protocol was approved by the Ethics review board of the Third Military Medical University. The effluent samples were collected from human donor's hearts for transplantation. Five donors were brain dead due to car accident, their respiration was maintained by mechanical ventilation and hemodynamics was stabilized by minimum doses of catecholamine. The donors with normal cardiac function were brought into our study. The donors with sustained (> 5 min) profound hypotension (≤ 50 mmHg on systolic pressure), cardiac arrest, intracardiac injection and history of heart disease or heart injury due to thoracic trauma, diabetes mellitus, hypertension, coronary arterial disease were excluded. CK-MB and cTnI in serum were measured before the operation for harvesting the heart. Clinical characteristics are shown in Table 1.

**Table 1:** Clinical information of the five heart donors

**No**.	Sex	Age	Blood type	CK-MB (ng/mL)	cTNI (ng/mL)
1	M	36	B	12	--
2	M	28	AB	22	--
3	M	32	O	16	--
4	F	35	A	11	--
5	F	32	B	13	--

The operation of the donor heart dissection was performed with a standard procedure. Briefly, the patients were anesthetized with isoflurane, intubated and ventilated with 100% oxygen. The heart and great vessels were exposed after sternotomy. Heparin at 3 mg/kg was injected intravenously for systemic anticoagulation. After a cardioplegic cannula was placed into the ascending aorta, one liter of a hypothermic (4°C) hyperkalemic crystalloid solution (composition in mmol/L: Na + 127, K + 20, Mg2+ 8, Cl- 20, SO4 - 8, HCO3 - 20, pH 7.9) was infused into the aortic root to achieve cardioplegic arrest. After cardioplegic infusion, the donor heart was extracted and placed in a basin containing 2000 ml cardioplegia solution at 4°C, The cardioplegic cannula and aortic cross-clamp were left in place to permit perfusion. Then, hypothermic cardioplegia was infused continually through the cardioplegic cannula until there was no blood in the effluent judged by eyes. A catheter was placed from inferior vena entrance into the exit of coronary sinus. The catheter was fixed and these open ends of superior and inferior vena, pulmonary arteries and veins were clamped. 200 ml hypothermic cardioplegia was infused into aortic root within 2 min and the effluent from the coronary sinus was collected into the 50 ml polypropylene tubes as the sample for the first time point. Finally, the heart was placed in a bag, and stored on ice. After 30-min ischemia, 200 ml cold cardioplegia was infused through the cardioplegic cannula, effluent was collected for the second time point. After ischemia for 60 min, effluent was collected again as the sample for the third time point. The effluent samples from each of the 5 hearts were mixed with protease inhibitors (1.67 mL of 100 mM NaN3, 2.5 mL of 11.5 mM PMSF) to avoid proteolysis and then cell debris and insoluble solids were removed by centrifugation at 10 000 × g for 15 min at 4°C. The precipitates were removed and then the supernatants were concentrated by using the Amicon Ultra-15 3 kDa MWCO centrifugal filters (Millipore, USA). Finally, the protein was stored at -80°C for further analysis.

### Removal of High-Abundance Proteins

All procedures of removing high-abundance proteins were performed according to manufacturer's instructions. Briefly, each effluent sample was thawed and centrifuged at 10 000 × g for 30 min at 4°C. Multiple affinity removal system 4.6 × 50 Hu-7 (5188-6409, Agilent Technologies) was used to remove high-abundance proteins from the effluent samples according to a standard liquid chromatography protocol. Briefly, the sample was diluted three times with Buffer A and centrifuged at 16 000 g for 10 min. The diluted sample was used for injection at a flow rate of 0.25 ml/min. The low-abundance protein fraction from 1.5 min to 4.5 min was collected into a 1.5 ml Eppendorf tube and store at 4°C for further analysis, and the high-abundance protein fraction was eluted with Buffer B at a flow rate of 1.0 ml/min, then the column was regenerated with buffer A.

### Protein concentrating and desalting

The low-abundance proteins were then concentrated and desalted by using the Amicon Ultra-15 10 kDa MWCO centrifugal filters (Millipore) according to manufacturer's instructions. Briefly, 10 ml of low-abundance fraction was filled into five spin concentrators with 10 kDa molecular weight cutoff. The samples were spun at 14 000 g for 30 min at 4°C, the filtrate was discarded, the concentrated protein was kept, and then the concentrator was refilled with an appropriate buffer (8 M urea) and spun again. After repeating this procedure three times, the sample was collected. Protein content was determined by using Bradford assay kit (Thermo, USA) and the sample was aliquoted into 40 ug fractions.

### In-solution tryptic digestion

In-solution tryptic digestion was performed according to the standard protocol provided by Agilent Technologies. Briefly, the Eppendorf tube contained 40 ug protein was added to 10 μl of 10 mM dithiothreitol (DTT) and incubated for 1 h at 56°C. After 20 μl of 20 mM iodoacetamide (IAM) was added, the Eppendorf tube was placed in dark for 1 h at room temperature. Then, 10 μl of 10 mM DTT was added again to quench the excess IAM for 1 h at 37°C. Protein was digested by 40 μl trypsin (12.5 ng/μL) (Sequencing Grade, Promega, USA) for 12 h at 37°C. Finally, the reaction was terminated by adding 2.5 μl formic acid.

### High performance liquid chromatography-Chip-Mass spectrometry (HPLC-Chip-MS) system analysis

The sample was analyzed on an Agilent 1200 HPLC and 6330 Ion Trap system (Agilent technologies, USA) as described previously [8-10]. 1 μl of digests (400 ng) were injected on a Zorbax chip composed of an enrichment column (560.3 mm, 5 mm particles) and a Zorbax 300SB C18 (75 μm × 150 mm, 3.5 mm particles) analytical column. The mobile phase for both capillary pump and nanopump consisted of 0.1% formic acid in distilled water (A) and 0.1% formic acid in 90/10 acetonitrile/distilled water (B). The flow rate for the capillary pump was held constant at 4 μl/min in 3% B (isocratic) while the flow rate for the nanopump was 0.3 μl/min, following a gradient of 3 - 75% B in 70 min. The mass spectrum was operated in chip positive ionization mode, with voltage at 4 KV, drying gas temperature at 325°C and drying gas flow at 6 L/min. In-source voltage was set at 1850 V, capillary exit at 96.4 V, skimmer at 40 V, end plate offset at -500 V. Automatic MS/MS in a data-dependent manner was acquired in enhanced mode at *m*/*z *200 - 1600. Due to statistical fluctuations of peptide precursor selection during MS/MS acquisition, three LC-MS/MS assays were run with each sample in order to be able to do a proper proteomic comparison.

### Enzyme-linked immunosorbent assays (ELISA)

The concentration of three interested proteins identified by MS was quantitatively determinated with phosphoglycerate mutase 2(PGAM2) human ELISA kit (Cusabio biotech CO., LTD, China), L-lactate dehydrogenase B(LDHB) human ELISA kit and titin human ELISA kit (Uscn Life Science Inc, China) according to the manufacturer's instructions in a blinded manner. Optical densities were measured at 450 nm by an eight-channel spectrophotometer. The concentration of LDHB, PGAM2 and titin was calculated using respective assay standard curves. The results were represented as mean ± SD.

### Data analysis and statistics

The MS/MS data were searched automatically against the international protein index (IPI)human database [11] using the Spectrum Mill Proteomics Workbench software (RevA.03.03, Agilent, USA). Only peptides with Spectrum Mill score more than 8 and Spectrum Mill Scored Peak Intensity(SPI) > 70% were considered positives. The confidence of all identified proteins must have more than 95%. In addition, protein with *p*-value < 0.05 was considered as significant. After each protein was identified, the relative abundance of protein in the sample was quantified by recording the mean of the peak intensities(MPI) of the component peptides [12]. The quantitative results were analyzed by one way analysis of variance followed by the Tukey test after confirmation of normal distribution of the data (data are presented as means ± SD) or by Kruskal-Wallis analysis of variance on ranks followed by the Dunn's test when the data are not normally distributed. A *P *≤ 0.05 was accepted as significant. All statistical analyses were performed via the SigmaStat software (Systat Software, Inc., Point Richmond, CA) [13]. Gene Ontology (GO) analysis was done by using Expression Analysis Systematic Explorer (EASE) software [14]. A protein-protein interaction network on metabolism was done by downloading pathway data from Kyoto Encyclopedia of Genes and Genomes(KEGG) database, and then enzyme-enzyme interaction (ECrel) and protein-protein interaction (PPrel) was analyzed by KEGGSOAP software [15]. Finally, the network of relationship between protein to protein was built and brought forth by Medusa software [16]. The network was graphically visualized as nodes (proteins) and edges (the relationships between proteins).

## Results and discussion

### Clinical data and high-abundant protein removal

**Figure 1:**
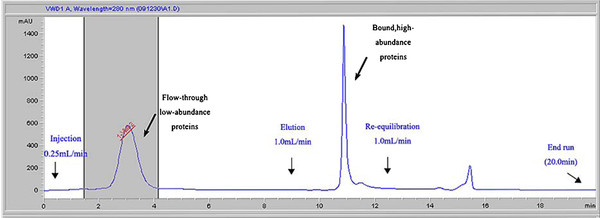
**Chromatogram of the affinity removal of high-abundant proteins from the effluent**. Injections of 75 ul of 4 × diluted effluent in buffer A were made on a Multiple Affinity Removal Column (4.6 × 50 mm) at a flow rate of 0.25 ml/min in buffer A. Flow-through fractions were collected from 1.5 - 4.5 min. The bound fraction was eluted with buffer B at a flow rate of 1.0 ml/min for 3.5 min.

Since all hearts in our study did not have obvious cardiac injury history, serum cTnI in all patients was negative and serum CK-MB was within normal limits. Table 1 lists the clinical characteristics. Proteomic analysis of isolated perfused organ effluent with less blood is a novel model for protein biomarker discovery [7]. However, the residual high-abundant plasma proteins such as albumin, immunoglobulin, transferrin, haptoglobin and antitrypsin were still present in the effluent. We removed these residual plasma proteins from effluents by using Multiple Affinity Removal Column Hu-6HC before proteomic analysis, and finally none of them was identified by HPLC-Chip-MS system. This process made the detection of low abundant proteins easier. The chromatogram of the affinity removal of high-abundant proteins is shown in Figure 1.

### Protein identification by HPLC-Chip-MS system

**Figure 2:**
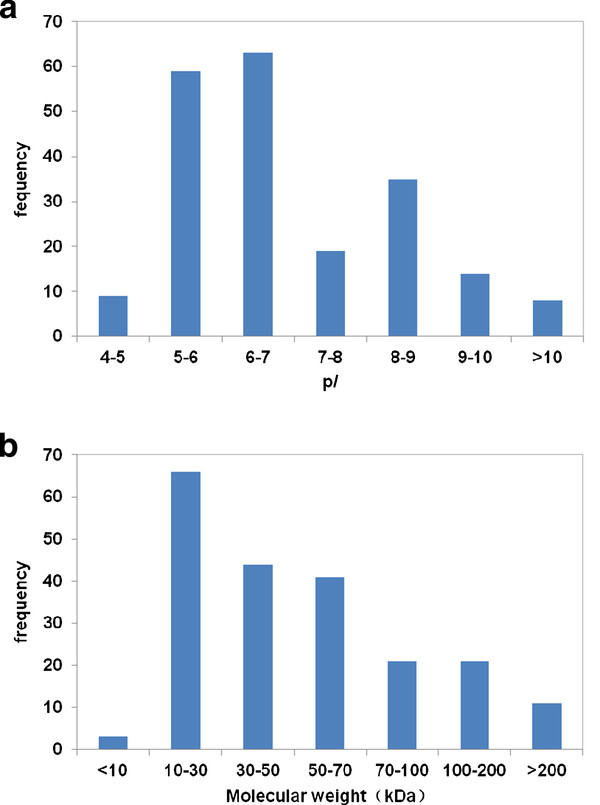
**Molecular weight and p*I *distribution of proteins identified consistently in five hearts' effluents**. **A**. Presented was p*I *distribution of identified proteins. **B**. Presented was molecular masses distribution of identified proteins.

**Figure 3:**
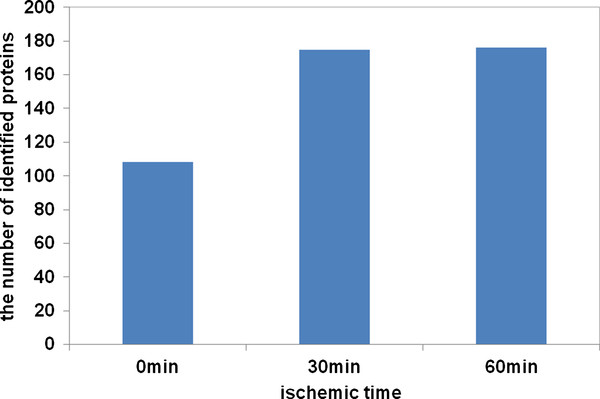
**The relationship between the number of proteins identified by HPLC-Chip-MS system and ischemic time**. Longer durations of ischemia increased the number of identified proteins.107 proteins were identified at the beginning of ischemia, 174 and 175 proteins were identified respectively at 30 min and 60 min after the onset of ischemia.

Biomarkers are often in low abundance and very difficult to be identified by using conventional proteomic approaches. HPLC-Chip-MS method used in our study has been proved to be a valuable tool for identifying protein biomarkers with high sensitivity and reproducibility, even for proteins expressed in very low abundances [17]. Total 618 proteins were identified and among of them, highly reliable proteins are 196 (listed its source, function, and molecular weight in Additional file 1). A 29 kDa unknown protein (IPI00877674) was identified and expressed mostly at myocardium by immunohistochemistry (data not shown). The molecular weight (Mr) and isoelectric point(p*I*)distribution of highly reliable proteins are shown in Figure 2. These proteins all showed a fairly wide p*I *(4.09-11.7) distribution (Figure 2A), and most of them(73.9%) are smaller than 70 kDa (Figure 2B). Longer durations of ischemia, increased the number of identified proteins (Figure 3). 107 proteins were identified at the beginning of ischemia, 174 and 175 proteins were identified, respectively, at 30 min and 60 min after the onset of ischemia (listed in Additional file 2).

With exception of cardiac troponin I and T, many known clinic markers for myocardial ischemia and several potential new biomarkers were detected in our study, such as aspartate aminotransferase (ASA), CK-MB, heart-type fatty acid-binding protein (H-FABP), lactate dehydrogenase B (LDHB), and myohemoglobin (MB) (Table 2). However, we did not detect ischemia modified albumin in the effluents, the biomarker for early myocardial ischemia, nor did Koomen *et al *in a rat model [7]. This protein were not detected possibly because of the removal of albumin by multiple affinity removal system in our study. In addition, five additional proteins specific to heart or muscle were identified: cardiac alpha actin, isoform alpha-enolase of alpha-enolase (ENO1), phosphoglycerate mutase 2 (PGAM2), isoform M1 of pyruvate kinase isozymes M1/M2(PKM2) and isoform 8 of Titin. Neither cTnI nor cTnT were detected possibly because of the lower level of cTnI and cTnT release with mild ischemic injury of myocardium, their late release, or few detectable peptides for them after use of tryptic digestion. Four proteins expressed abundantly in heart were identified including Cysteine-rich protein 2,S100 calcium binding protein A1, 14 kDa phosphohistidine phosphatase and mesenchymal stem cell protein DSC92. In additional, those identified proteins including Glucose-6-phosphate isomerase (GPI), Creatine kinase M-type (CKM), Malate dehydrogenase (MDH1), Creatine kinase B-type (CKB), (heart-type)Fatty acid-binding protein (FABP3), Glycerol-3-phosphate dehydrogenase [NAD+](GPD1), PGAM2, MB, Superoxide dismutase(SOD1), Adenylate kinase 1(AK1), Isoform 1 of Triosephosphate isomerase(TPI1), Phosphatidylethanolamine-binding protein 1(PEBP1), Glyceraldehyde-3-phosphate dehydrogenase (GAPDH), Aspartate aminotransferase,(GOT1) and Four and a half LIM domains 1 variant(FHL1) were also detected in coronary sinus plasma of planned myocardial infarction patients by Addona *et al *[18].

**Table 2:** Part of the identified proteins that may be interesting in potential biomarker discovery of early ischemia in myocardium

Protein name	Function	Distinct peptide	MW(Da)	Organ specific	Clinical marker
Isoform M1 of Pyruvate kinase isozymes M1/M2	metabolism	12	58,062.4	yes	yes
Actin, alpha cardiac muscle 1	cytoskeleton	8	42,019.2	yes	
Aspartate aminotransferase 1	metabolism (amino acid)	6	46,247.7		yes
Creatine kinase M-type	energ transduction	15	43,101.4	yes	yes
Heart -fatty acid-binding protein,	lipid transport	9	14,858.1	yes	yes
Glyceraldehyde-3-phosphate dehydrogenase	metabolism (glycolysis)	14	36,053.4		
L-lactate dehydrogenase B chain	metabolism (glycolysis)	14	36,638.7	yes	yes
Myoglobin	oxygen transport	17	17,183.9	yes	yes
Isoform 1 of Triosephosphate isomerase	metabolism (glycolysis)	12	30,791.2		
Isoform 8 of Titin	cytoskeletal activity	4	3829,893.5	yes	
Glucose-6-phosphate isomerase	metabolism (glycolysis)	5	63147.5		
Isoform 1 of Gelsolin	cytoskeletal activity	9	85697.9		
Phosphoglycerate mutase 2	metabolism (glycolysis)	2	28766.3	yes	
Adipocyte -fatty acid-binding protein	lipid transport	9	14719		
Adenylate kinase 1	ATP regeneration	5	23410.9		
Creatine kinase B-type	kinase activity	2	42644.5		
Four and a half LIM domains 1 variant	protein binding	5	33578.9		
Malate dehydrogenase, cytoplasmic	metabolism (glycolysis)	12	36426.3		
Phosphatidylethanolamine-binding protein 1	protease inhibitor	8	21056.9		
PGM1 65 kDa protein	magnesium ion binding	3	64560.9		
Superoxide dismutase	oxidoreductase	1	15935.8		

**Figure 4:**
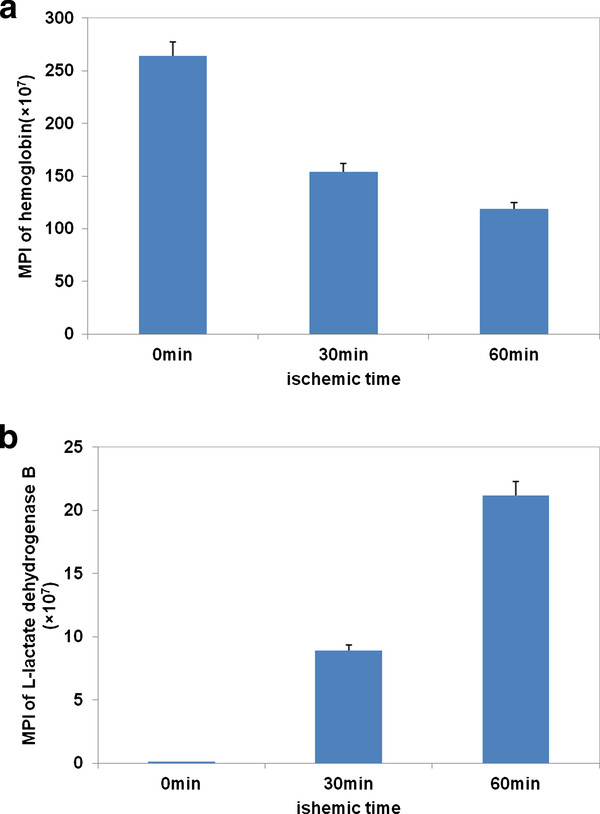
**The relationship between the mean of peak intensities(MPI) and ischemic time. A**. Presented was the relationship between the mean of peak intensities and ischemic time in hemoglobin. **B**. Presented was the relationship between the mean of peak intensities and ischemic time in L-lactate dehydrogenase B.

**Figure 5:**
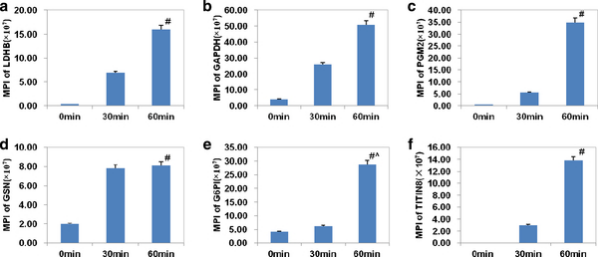
**Six proteins released significantly into the effluents during myocardial ischemia by quantitative analysis of the mean of the peak intensities (MPI)**. A. The MPI of L-lactate dehydrogenase B (LDHB). **B**. The MPI of glyceraldehyde-3-phosphate dehydrogenase (GAPDH). **C**. The MPI of glucose-6-phosphate isomerase (GPI). **D**. The MPI of phosphoglycerate mutase 2 (PGAM2). **E**. The MPI of gelsolin. **F**. The MPI of isoform 8 of titin. Among them, LDHB, GAPDH, GPI and PGAM2 are glycolytic enzymes. Gelsolin and isoform 8 of titin are targets of matrix metalloproteinase (MMP). Value are expressed as means ± SEM for each group. #*p *< 0.05 represents a significant difference in the ischemia for 30 min and ischemia for 60 min group compared with the ischemia for 0 min group; ^*p *< 0.05 represents a significant difference in the ischemia for 60 min group compared with the ischemia for 30 min group.

The relative protein levels were compared by the Spectrum Mill software according to MPI of the component peptides. Trends in MPI of proteins matched by database searches can be used to determine which proteins were released from the tissue. For examples, the MPI of the plasma proteins, such as hemoglobin, followed a gradually decreasing trend as they were removed away from the blood vessel by perfusion with crystalloid solution (Figure 4A). The MPI of LDHB indicates the increasing trend for proteins released from the heart (Figure 4B). The quantitative results showed that there were six proteins released from the heart when ischemic time was increasing, including LDHB, glyceraldehyde-3-phosphate dehydrogenase (GAPDH), glucose-6-phosphate isomerase (GPI), PGAM2, gelsolin and isoform 8 of titin (Figure 5). Four of these six proteins are glycolytic enzymes, including LDHB, GAPDH, GPI, PGAM2, and two of them (gelsolin and isoform 8 of titin) are targets of matrix metalloproteinase (MMP), which will be activated during myocardial ischemia/reperfusion injury [19-22]. Isoform 8 of titin is specifically expressed in cardiac muscle with a large molecular weight (~3800 kDa) and is very important in the contraction of striated muscle tissues. It is unclear how isoform 8 of titin was released into the effluent during myocardial ischemia. Ali *et al *reported that titin was degraded by MMP-2 during myocardial ischemia/reperfusion injury [21]. Thus, those degradation products of titin could be released from myocardial cells and be detected in the effluent.

**Figure 6:**
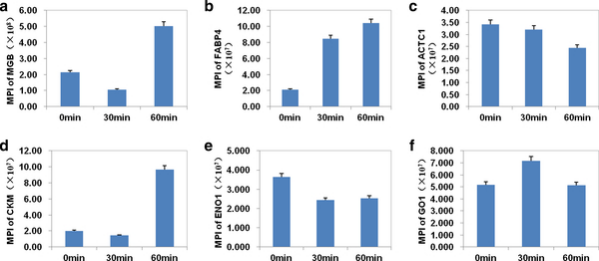
**Six proteins specific to heart had little change in the effluents during myocardial ischemia by quantitative analysis of the mean of the peak intensities (MPI)**. **A**. The MPI of MB.**B**. The MPI of H-FABP. **C**. The MPI of alpha actin. **D**. The MPI of CK-M. **E**. The MPI of ENO1. **F**. The MPI of PKM2.

However, the level of six proteins specific to heart had little change in the effluent when the myocardium was undergoing ischemia. These proteins are MB, H-FABP, alpha actin, CK-M, ENO1, PKM2 (Figure 6). But many of them showed a rising trend. On the hand, those proteins may not be released into the effluent significantly because of a mild ischemic injury under hypothermia. Under this condition, myocardium may be undergone a reversible cellular damage, such as myocardial stunning or hibernation, rather than an irreversible cellular damage, such as myocardial infarction [23,24]. These markers for myocardial infarction detected in the effluent, including MB and CK-MB may be released into the blood due to myocardial injury by surgery procedure or ischemia before our measurement. On the other hand, the number of samples is not big enough in this study. Further researches will be needed to test this issue.

### Comfirmation of protein expression by ELISA

**Figure 7:**
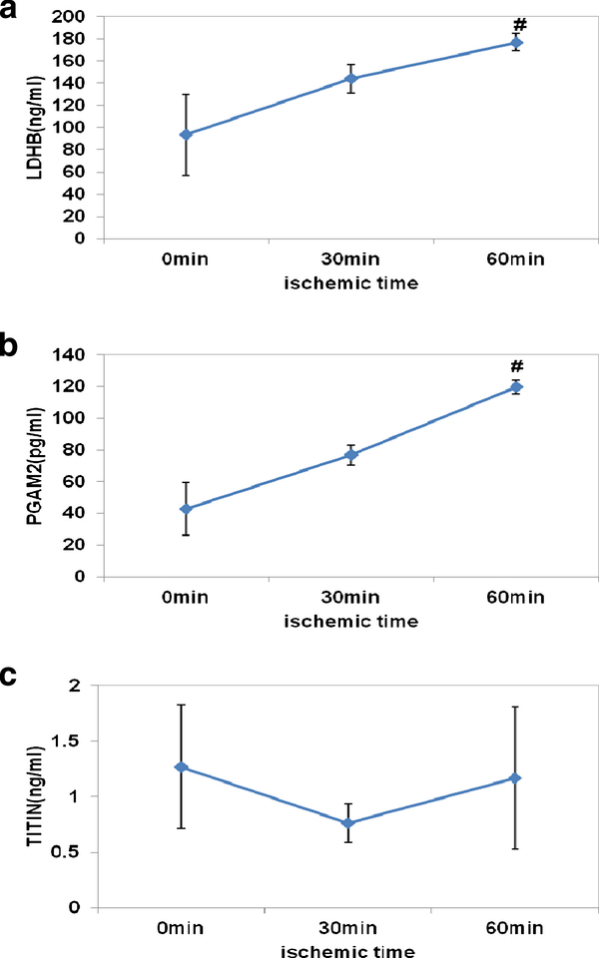
**Comfirmation of protein expression by ELISA**. The concentration of three interesting proteins was quantitatively determinated with ELISA kits(n = 5). **A**. The mean concentration of LDHB.**B**. The mean concentration of PGAM2. **C**. The mean concentration of Titin. Value are expressed as means ± SEM for each group. #*p *< 0.05 represents a significant difference in the ischemia for 60 min group compared with the ischemia for 0 min group.

To quantify the expression of some identified proteins, samples were further examined by ELISA. The concentrations of LDHB and PGAM2 showed an increasing trend in effluent samples of perfused heart when ischemic time was extending. The mean concentrations of LDHB at ischemia for 30 min and at ischemia for 60 min were approximately 1.5-fold higher and 1.9-fold higher than at ischemia for 0 min respectively (Figure 7A), whereas the mean concentrations of PGAM2 were approximately 1.8-fold higher at ischemia for 30 min and 2.8-fold higher at ischemia for 60 min than at ischemia for 0 min(Figure 7B). But the mean concentration of Titin was almost no change while ischemic time was increasing (Figure 7C). It's unclear what happened on it. Maybe the antibody of titin in the ELISA kit is unable to combine with isoform 8 of titin specifically, or the antigenic epitope for anti-titin was destroyed after titin was degraded by MMP-2 and released from myocardial cells [21]. As a biomarker of myocardial infarction, LDHB was identified by MS in our study, so did by Koomen JM *et al *with the same perfused heart effluent model as ours [7]. HPLC-Chip-MS method is more sensitive than clinical assay and the component of the effluent sample is much simpler than that of plasma, so LDHB could be detected sensitively in our study even if a few of cardiac cells are damaged by ischemia.

### GO analysis

**Figure 8:**
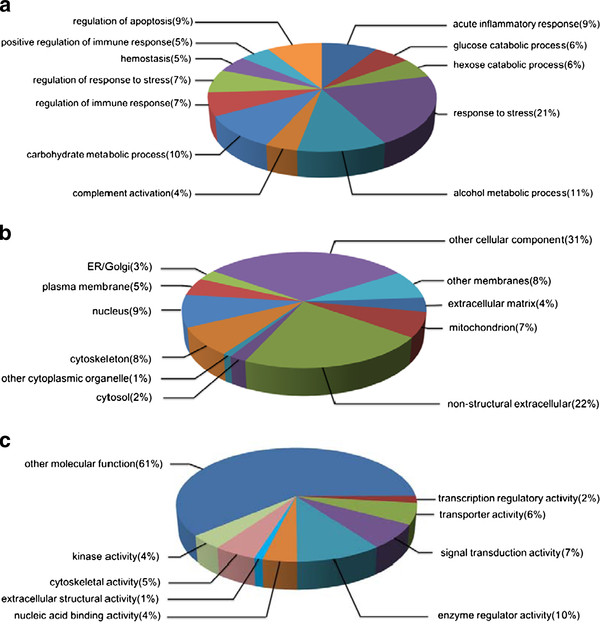
**Gene Ontology classification generated by Software Tool for Researching Annotations of Proteins**. Pie charts showing the classification of the identified proteins according to their biological functions, cellular component and molecular function. **A**. The biological functions of the identified proteins were diverse. However, a large percentage of them are related to the heart function during ischemia and reperfusion: ATP regeneration, metabolism (in particular glycolysis), oxidative stress response and protective proteins. 33% of the identified proteins were involved in the metabolic process for carbohydrate (22%) and alcohol (11%), 28% were involved in stress process and 9% were respectively involved in regulation of apoptosis and acute inflammatory response (Figure 7A). **B**. Of the 196 unique proteins identified in these experiments, only 31% were plasma proteins. The remainders were from the cellular organelles (28%) as well as a variety of other cellular components(41%). **C**. The molecular functions of the identified proteins were diverse, but a large percentage could be related to enzyme regulation (10%), signal transduction (7%), transporter (6%), cytoskeletal activity (5%).

The biological functions of the identified proteins were diverse. However, a large percentage could be related to the heart function during ischemia and reperfusion: ATP regeneration, metabolism (in particular glycolysis), oxidative stress response and protective proteins. 33% of the identified proteins were involved in the metabolic process for carbohydrate (22%) and alcohol (11%), 28% were involved in stress process and 9% were respectively involved in regulation of apoptosis and acute inflammatory response (Figure 8A). Only 31% were plasma proteins; the rest were from from a variety of cellular organelles(28%) and the other cellular components(41%) (Figure 8B). A large percentage of identified proteins could be related to enzyme regulation (10%), signal transduction (7%), transporter (6%) and cytoskeletal activity (5%)(Figure 8C).

### Gene network analysis for metabolism

**Figure 9:**
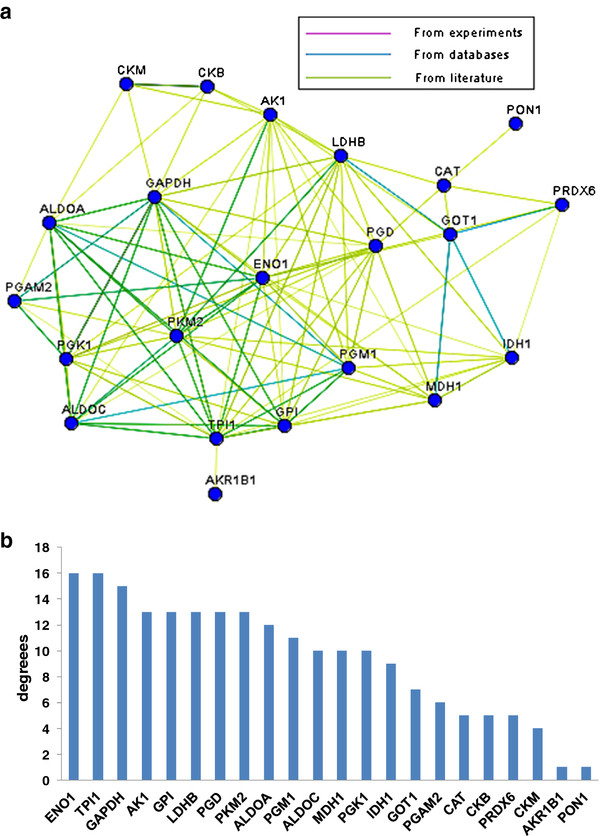
**The protein-protein interaction network on myocardial metabolism. A**. protein-protein interaction network on metabolism was done by downloading pathway data from Kyoto Encyclopedia of Genes and Genomes(KEGG) database, then enzyme-enzyme interaction (ECrel) and protein-protein interaction (PPrel) was analyzed by KEGGSOAP software. Finally, the network of relationship among proteins was built and brought forth by Medusa software. The network was graphically visualized as nodes and edges. Pink lines indicate connections confirmed experimentally by other researches, blue lines indicate connections derived from databases and yellow lines indicate connections compiled from co-citation data from literature mining PubMed abstracts. Interestingly, about 55% of those proteins showing in the network are involved in glycolysis. **B**. The number of connections of each protein was calculated. ENO1, TPI1 and GAPDH are central "functional hubs" in the map with more connections than other proteins. They are all important glycolytic enzymes in myocardium.

After myocardial ischemia, more than twenty proteins involving in metabolism were identified by HPLC-Chip-MS system. To evaluate the eventual correlation existing between these identified proteins, the network analysis on metabolism was performed by KEGGSOAP software. Known interactions coming from KEGG interaction databases were graphically visualized as nodes and edges. The map is shown in Figure 9A. Interestingly, about 55% of those proteins showing in the network are involved in glycolysis. The number of connections of each protein was calculated. ENO1, TPI1 and GAPDH are central "functional hubs" in the map with more connections than other proteins, which have been extensively reported to take part in glycolysis in myocardium. The degree of connection is shown in Figure 8B. In order to adapt to oxygen deprivation, myocardial cells will reprogram their metabolism induced by the PHD/HIF system and glycolytic enzymes will be expressed during ischemia [25]. Thus, many glycolytic enzymes will be expressed and could be released from myocardial cells during early ischemia, such as L-LDH, GAPDH, GPI and PGAM2. Type-M of PGAM2 is specifically expressed in muscles and could be a potential biomarker for early myocardial ischemia.

## Conclusion

In this study, we analyzed low-abundance proteins from effluents of perfused human hearts after mild ischemic injury by HPLC-Chip-MS system. Many known clinic markers for myocardial ischemia, proteins specific to heart and glycolytic enzymes were among the proteins identified. Among them, four glycolytic enzymes, L-LDH, GAPDH, GPI, PGAM2, and two targets of MMP, gelsolin and isoform 8 of titin, are significantly released into the effluent during myocardial ischemia. They are potential biomarkers for mild ischemic injury in myocardium, especially isoform 8 of titin and M-type of PGAM2, which are more specific for cardiac tissue. Furthermore, the results of gene network analysis for metabolism show glycolysis is one of the important conversions during early ischemia in myocardium. This finding may provide new insight into pathology and biology of myocardial ischemia, and potential diagnostic and therapeutic biomarkers.

## Abbreviations

IHD: Ischemic heart disease; CK-MB: Creatine kinase muscle/brain isoform; CKM: Creatine kinase M-type; CKB: Creatine kinase B-type; cTnT: Cardiac troponin T; cTnI: Cardiac troponin I; ACS: Acute coronary syndromes; p*I*: Isoelectric point; MW: Molecular weight; MWCO: Molecular weight cutoff; DTT: Dithiothreitol; IMA: Iodoacetamide; HPLC: High performance liquid chromatography; MDH1: Malate dehydrogenase; MS: Mass spectrometry; IPI: International protein index; SPI: Scored peak intensity; EASE: Expression analysis systematic explorer; LDH: Lactate dehydrogenase; LDHB: Lactate dehydrogenase B; ASA: Aspartate aminotransferase; MB: Myohemoglobin; PKM2: Isoform M1 of pyruvate kinase isozymes M1/M2; GPI: Glucose-6-phosphate isomerase; H-FABP: Heart-type fatty acid-binding protein; GAPDH: Glyceraldehyde-3-phosphate dehydrogenase; TPI1: Isoform 1 of triosephosphate isomerase; PGAM2: Phosphoglycerate mutase 2; ENO1: Isoform alpha-enolase of alpha-enolase; MMP: Matrix metalloproteinase; HIF: Hypoxia-inducible factor; PHD: Prolyl hydroxylase; MW: Molecular weight; FDR: False discovery rate; ELISA: Enzyme-linked immunosorbent assays; GO: Gene ontology; KEGG: Kyoto encyclopedia of genes and genomes; MPI: Mean of the peak intensities.

## Competing interests

The authors declare that they have no competing interests.

## Authors' contributions

HL conceived the study, performed MS raw data processing, bioinformatics analysis and drafted the manuscript. JL contributed to experiments for sample preparation, participated in MS raw data processing, and mass spectrometry analysis. YW performed in solution digestion, HPLC-Chip-MS analysis and protein identification. TY contributed to overall design of this study. All authors read and approved the final manuscript.

## Supplementary Material

Additional file 1 **Total 196 proteins(2003-2007)**. Total 196 high reliable unique proteins were listed with its source, function, and molecular weight. Click here for file

Additional file 2 **Proteins identified by different ischemic time(2003-2007)**. 107 proteins were identified at the beginning of ischemia, 174 and 175 proteins were identified respectively at ischemia for 30 min and ischemia for 60 min. Click here for file
